# FLIM-Based Intracellular and Extracellular pH Measurements Using Genetically Encoded pH Sensor

**DOI:** 10.3390/bios11090340

**Published:** 2021-09-15

**Authors:** Alexander S. Goryashchenko, Alexey A. Pakhomov, Anastasia V. Ryabova, Igor D. Romanishkin, Eugene G. Maksimov, Alexander N. Orsa, Oxana V. Serova, Andrey A. Mozhaev, Margarita A. Maksimova, Vladimir I. Martynov, Alexander G. Petrenko, Igor E. Deyev

**Affiliations:** 1 Shemyakin-Ovchinnikov Institute of Bioorganic Chemistry of the RAS, 117997 Moscow, Russia; alpah@mail.ru (A.A.P.); Saniaorsa@gmail.com (A.N.O.); oxana.serova@gmail.com (O.V.S.); a.a.mozhaev@gmail.com (A.A.M.); ritamax2000@yandex.ru (M.A.M.); vimart@list.ru (V.I.M.); petrenkoag@gmail.com (A.G.P.); deyevie@gmail.com (I.E.D.); 2 A. N. Nesmeyanov Institute of Organoelement Compounds of the RAS, 119991 Moscow, Russia; 3 Prokhorov General Physics Institute of the RAS, 119991 Moscow, Russia; nastya.ryabova@gmail.com (A.V.R.); Igor.Romanishkin@gmail.com (I.D.R.); 4 Department of Biology, M.V. Lomonosov Moscow State University, 119234 Moscow, Russia; emaksimoff@yandex.ru; 5 A.V. Shubnikov Institute of Crystallography of Federal Scientific Research Centre “Crystallography and Photonics” of Russian Academy of Sciences, 119991 Moscow, Russia; 6 Sirius University of Science and Technology, 354340 Sochi, Russia

**Keywords:** GFP, SypHer3s, SypHerExtra, fluorescent proteins, pH, pH sensor, intracellular pH, extracellular pH, FLIM

## Abstract

The determination of pH in live cells and tissues is of high importance in physiology and cell biology. In this report, we outline the process of the creation of SypHerExtra, a genetically encoded fluorescent sensor that is capable of measuring extracellular media pH in a mildly alkaline range. SypHerExtra is a protein created by fusing the previously described pH sensor SypHer3s with the neurexin transmembrane domain that targets its expression to the cytoplasmic membrane. We showed that with excitation at 445 nm, the fluorescence lifetime of both SypHer3s and SypHerExtra strongly depend on pH. Using FLIM microscopy in live eukaryotic cells, we demonstrated that SypHerExtra can be successfully used to determine extracellular pH, while SypHer3s can be applied to measure intracellular pH. Thus, these two sensors are suitable for quantitative measurements using the FLIM method, to determine intracellular and extracellular pH in a range from pH 7.5 to 9.5 in different biological systems.

## 1. Introduction

The measurement of pH in an organism or in cellular compartments has various applications in cell biology and medicine. In live systems, the acid–base balance controls many biochemical processes, and pH disturbances largely affect cell growth, cell division, and its functions [1]. pH dysregulation is associated with multiple human diseases, such as kidney failure, Alzheimer’s disease, and cancer [2,3,4,5]. It is also known that even small pH disturbances can alter prohormone processing [6,7]. Thus, the task of accurate pH measurement in living systems, although technically quite challenging, is extremely important, and over the past twenty years, the development of fluorescent probes was aimed to solve this problem.

The diversity of synthetic and genetically encoded fluorescent sensors has increased in recent years, and thus has given us powerful tools for determining pH in living cells [8]. Genetically encoded sensors have many benefits and are potentially suitable for targeting tissues and cells and for being expressed in transgenic animals [9,10]. Most of the currently available pH sensors [11,12] have a pKa that lies in the acidic to neutral pH range, and there are only a few that have an alkaline pKa. The best sensors out of those with an alkaline pKa are the ratiometric sensors deGFP1 (pKa 8.0) [13], SypHer2 (pKa 8.1) [14], and SypHer3s (pKa 7.8) [15]. All of them were successfully used to measure intracellular pH. 

SypHer3s is a genetically encoded fluorescent pH probe with a ratiometric readout from pH 5.5 to 10.0, and an improved brightness compared to its predecessors SypHer and SypHer2 [15]. Originally, SypHer was generated by introducing a C199S mutation to the hydrogen peroxide sensor HyPer [16]. This mutation makes HyPer insensitive to hydrogen peroxide while preserving its sensitivity to pH variations.

Fluorescent pH sensors have a ratiometric or intensiometric readout. Ratiometric readout means that the sensor’s analytical signal is the ratio of its fluorescence intensity at different wavelengths. Such readout provides quantitative measurements and has no potential artifacts that might arise due to cell movement or changes in the cell’s shape. While a ratiometric readout is good enough in the case of in cellulo measurements, the different depth of wavelength penetration through the tissues of living organisms may result in incorrect pH measurements inside the organs or tissues. To solve this problem, the fluorescence lifetime imaging microscopy (FLIM) technique could be used for the readout of the probe response [17,18]. In this study, we created the fluorescent sensor SypHerExtra, which is capable of measuring extracellular pH in a mildly alkaline range, and showed that it is suitable for the quantitative monitoring of extracellular pH by FLIM.

## 2. Materials and Methods

### 2.1. Plasmid Construction

Chimera construct pST-SH3-Nx based on pSecTag 2B vector with IgΚ leader peptide, which was fused with SypHer3 and rat neurexin transmembrane part, was obtained using PCR cloning strategy. Eukaryotic pC1-SypHer3s vector for cytoplasmic SypHer3s expression was kindly provided by Y.G. Ermakova, IBCh RAS. This vector was used as a template for PCR with 5′-AAAGGATCCATGTCCGGACCGCTGCACATT-3′ and 5′-TTTGAATTCCGCTAACCGCCTGTTTTAAAACTTTATC-3′ primers to produce SypHer3s cDNA. This coding sequence was cloned into the pSecTag vector using EcoRI and BamHI restriction sites. As a transmembrane linker that connects SypHer3s to a cell membrane we used a region of α-neurexin protein containing 146 amino acids from its extracellular part, 21 amino acids from the transmembrane domain and 35 amino acids from the cytoplasmic domain. cDNA of this linker was produced by PCR with pcDNA-Nx(SS6-)-HA plasmid [19] as a template, forward primer 5′-GCCGAATTCAGAAAGTTCTGAATATGGCGGCAGA-3′ and reverse primer 5′-GCGCTCGAGGACATAATACTCCTTATCCTTGTTC-3′. Obtained linker domain cDNA was inserted into the pSecTag-SypHer3s vector using EcoRI and XhoI restriction sites. The structure of the obtained vector was confirmed by sequencing.

### 2.2. Determination of the SypHerExtra Sensor Localization

To be sure that obtained SypHerExtra sensor has membrane localization, immunocytochemistry technique was carried out. Twelve millimeter P231.1 cover glasses (Menzel-Gläser, Braunschweig, Germany) were placed into a 24-well culture dish and treated with poly-L-lysine (Sigma-Aldrich, St. Louis, MO, USA). Then HEK293 and HeLa cells were seeded onto this culture dish and grown in Dulbecco’s modified Eagle medium (DMEM) supplemented with 10% fetal bovine serum (Hyclone, Logan, UT, USA), 1% penicillin/streptomycin, and 2 mM L-glutamine. Transfection with pST-SH3-Nx plasmid was carried out using Unifectin-56 (Unifect Group, Moscow, Russia) reagent, according to manufacturer’s protocol. Two days after transfection, the cells were washed with phosphate-buffered saline (PBS) and fixed with a mixture of methanol/acetone 1:1 *v/v* for 20 min at −20 °C. To prevent non-specific antibody adsorption, cells were incubated in PBS containing 2% bovine serum albumin for 3 h at room temperature. Then overnight incubation at 4 °C with anti-GFP primary antibodies (Evrogen, Moscow, Russia) dissolved in PBS at a ratio of 1:5000 was carried out. Unbound antibodies were washed off with PBS three times for 7 min, and then secondary goat antibodies conjugated with Cy3 against rabbit IgG were added (Jackson ImmunoResearch, Cambridge House, St. Thomas’ Place, Cambridgeshire, UK), dissolved in PBS at a ratio of 1:5000. After 1 h incubation at room temperature, unbound antibodies were washed off with PBS three times for 7 min followed by fixation of the cells on the cover slides using Glycergel mounting medium (Dako, Glostrup, Denmark). 

We also studied whether the SypHerExtra was endocytosed. HEK293 cells were co-transfected with pST-SH3-Nx and pmKate2-endo (Evrogen, Moscow, Russia) vectors and fixed on the cover slides using the protocol mentioned above. 

Cell imaging was carried out using Nikon Eclipse TE2000-E confocal microscope equipped with a 60x oil immersion objective. For SypHerExtra visualization, 488 nm laser excitation and 515/10 nm emission filter were used, while for Cy3-labeled antibodies and mKate2 visualization, 515 nm laser excitation and 650/30 nm emission filter were used.

### 2.3. Protein Preparation and UV/VIS Spectrophotometry

For protein expression, the cDNA encoding SypHer3s was cloned into the pQE-30 vector (providing N-terminal His-tag). *E. coli* (BL21(DE3) strain) cells were then transformed with this vector, followed by protein production in bacteria. The recombinant protein was further purified from the cells by immobilized metal affinity chromatography on Ni-NTA resin (Qiagen, Hilden, Germany) according to the manufacturer’s protocol and then dialyzed to the PBS. Absorption spectra of SypHer3s at different pH were collected in buffer STPCA (100 mM NaCl, 12.5 mM Tris, 12.5 mM sodium phosphate, 12.5 mM sodium acetate, 12.5 mM CAPS, pH 6.9) and titrated to desirable pH. Cary 50 Bio UV/VIS spectrophotometer (Varian, Palo Alto, CA, USA) was used to measure absorption spectra.

### 2.4. In Vitro Fluorescence Lifetime Measurements

In order to determine the relationship between the pH of a solution and the lifetime of SypHer3s, experiments were conducted on a series of buffers with pH values validated by standard methods. 

Purified SypHer3s samples were transferred into 500 mM Tris-HCl buffer with pH ranging from 6.5 to 9.5 with 0.5 increments, and then fluorescence lifetime measurements were performed using time-correlated single photon counting (TCSPC) system SimpleTau 140 (Becker&Hickl, Berlin, Germany) with 405 nm, 445 nm and 510 nm excitation lasers. Longpass filters (Thorlabs, Newton, NJ, USA) were used to filter out the excitation light. A ML-44 monochromator additionally filtered the emission (Solar, Minsk, Belarus). Fluorescence decay kinetics were analyzed with Becker&Hickl SPCImage software. Considering that at different pHs SypHer3s might be in two distinct conformational states, fluorescence decay kinetics were approximated using two-exponential model, and average fluorescence lifetimes were calculated. 

### 2.5. Cell Lines and Transfection

HEK 293 cells were grown on FluoroDish cell culture dishes with a 0.16-mm-thick glass bottom (WPI, Sarasota, FL, USA) in DMEM supplemented with 10% fetal bovine serum (Hyclone, Logan, UT, USA), 1% penicillin/streptomycin, and 2 mM L-glutamine and transfected with pC1-SypHer3s (for cytoplasmic SypHer3s) or pST-SH3-Nx (for SypHerExtra) plasmid using Unifectin-56 (Unifect Group, Moscow, Russia) reagent, according to manufacturer’s protocol. 

### 2.6. FLIM Measurements

To vary the intracellular pH of the HEK293 cells expressing cytoplasmic SypHer3s, nigericin ionophore was used. Two days after the transfection of the cells, the culture medium was replaced with 50 mM Tris-HCl buffers with pH from 6.5 to 9.5 containing 105 mM KCl, 1 mM MgCl2 and 10 µM nigericin according to [20] and, after 5 min of incubation, FLIM images of cells were obtained. 

Fluorescence lifetime measurements of the HEK293 cells expressing cytoplasmic SypHer3s were performed using Zeiss LSM-710 NLO confocal/multiphoton microscopy system consisting of the inverted Axio Observer (Zeiss, Jena, Germany) microscope, the femtosecond laser Chameleon Ultra II (80 MHz, 140 fs, tunable in 690–1060 nm, Coherent Inc., Santa Clara, CA, USA) used for excitation, and the HPM-100-07 hybrid GaAsP photodetector (Becker&Hickl, Berlin, Germany) connected to a non-descanned (NDD) port of the microscope. Appropriate dichroics and band-pass filters were used to collect SypHer3 fluorescence. The detection channel also contained a 760 nm short-pass filter (Zeiss, Germany) in the beam path to avoid the background excitation. SPC-150 TCSPC module (Becker&Hickl, Berlin, Germany) was synchronized with the laser pulses and the Zeiss LSM-710 scan head signals to collect the time-resolved fluorescence in TCSPC mode using SPCM acquisition software. SypHer3s fluorescence was excited at 890 nm laser wavelength through two-photon absorption. A 63x oil Plan-Apochromat NA 1.4 objective was used to acquire the FLIM images that consisted of 512 × 512 pixels with 1024 time channels. To compare different decay curves, we calculated the amplitude-weighted average fluorescence lifetime for each FLIM image. Average fluorescence lifetimes were calculated as follows: for each FLIM image, the lifetimes were calculated using two-exponential model (one for each chromophore state—neutral and anionic, each with its own lifetime) and Binning 3, then separate cells were chosen using area ROI and their lifetime distribution histograms were exported to the Origin Pro 9.1 software (OriginLab, Northampton, MA, USA). Next, nonlinear curve fit of the histograms was performed using GaussAmp model, and lifetimes corresponding to the histograms’ peaks were used as lifetimes of different cells on the frame. Based on these lifetime values, average fluorescence lifetime was calculated for each pH.

For SypHerExtra, the measurement protocol was the same except for the nigericin addition. To analyze obtained FLIM images, membrane regions of the cells were selected using linear ROI, and the average fluorescence lifetime for each pH was calculated as mentioned above. 

## 3. Results

**Figure 1:**
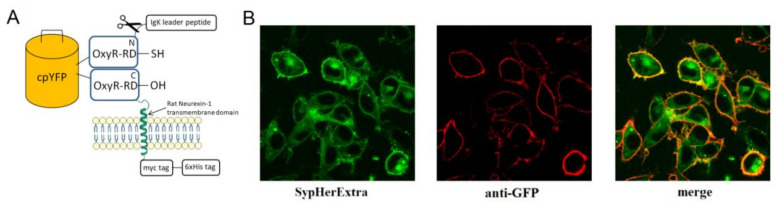
(**A**) Schematic representation of a genetically encoded construct SypHerExtra with extracellular expression and membrane anchoring; (**B**) fluorescent images of the fixed HEK293 cells expressing extracellular membrane-anchored SypHerExtra sensor (**left**) stained with anti-GFP antibodies (**center**). The merged image (**right**) indicates membrane-anchored SypHerExtra.

First, we generated a genetically encoded fluorescent sensor with membrane anchoring extracellular expression. For this, we made a chimera construct based on the pSecTag 2B vector with an IgΚ leader peptide, which was fused with SypHer3s and the neurexin transmembrane part for expression on the extracellular side of the cytoplasmic membrane (Figure 1A). We named it SypHerExtra. Transfection of HEK 293 or HeLa cells with this construct showed the expected localization of this chimera protein on the cell membrane (Figure 1B and Figure S1). 

**Figure 2:**
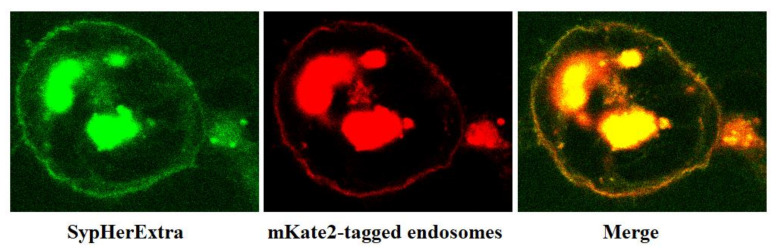
Fluorescent images of the fixed HEK293 cells expressing extracellular membrane-anchored SypHerExtra sensor (**left**) and mKate2 fluorescent protein targeted to endosomes (**center**). The merged image (**right**) indicates SypHerExtra colocalizated in endosomes.

In addition, we have studied the possible colocalization of SypHerExtra with endosomes. To visualize endosomes, we used the pmKate2-endo vector (Evrogen, Moscow, Russia) that encodes the far-red fluorescent protein mKate2, targeted to endosomes by human RhoB GTPase fused to the mKate2 C-terminus [21]. The co-transfection of HEK293 cells with pST-SH3-Nx and pmKate2-endo plasmids revealed that SypHerExtra colocalizates with endosomes (Figure 2). This would be a great opportunity to measure endo-lysosomal pH using the SypHerExtra sensor, but with a pKa of 7.8 [15], it is sensitive to pH values from neutral to mildly alkaline, while endosomal pH varies from 4.5 to 6.5 [22]. Nevertheless, the replacement of alkali-sensitive SypHer3s protein with an acid-sensitive one in the SypHerExtra construction could help to overcome this limitation.

**Figure 3:**
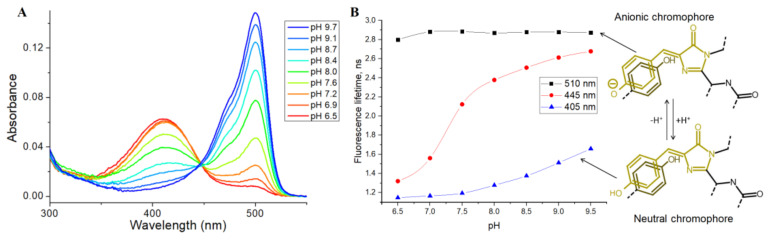
(**A**) Absorbance spectra of the SypHer3s at different pH; (**B**) pH dependence of the SypHer3s fluorescence lifetime in vitro using different excitation wavelengths—510 nm (black curve), 445 nm (red curve) and 405 nm (blue curve).

Then, we tested the applicability of SypHer3s as an FLIM pH sensor, by analyzing the pH dependence of its fluorescence lifetime, using the TCSPC (time-correlated single photon counting) method in the cuvette. SypHer3s has two peaks of light absorption in the visible region, at 410 and 500 nm, which correspond to neutral and anionic states of a chromophore, respectively (Figure 3A) [15].

Therefore, we used pulsed 405 and 510 nm lasers as a source of excitation light and measured the SypHer3s fluorescence lifetime at a pH ranging from 6.5 to 9.5. The detected fluorescence lifetimes at 405 and 510 nm excitation were almost independent of pH, and were found to be approximately 2.8 ns for the anionic chromophore (510 nm excitation) and 1.0 ns for the protonated chromophore (405 nm excitation) (Figure 3B). A small increase in the lifetime at high pH, at 405 nm excitation, is probably due to the absorption of an anionic chromophore at the tail of the main absorption peak of 500 nm. Next, we used a 445 nm pulsed laser that should effectively excite both the anionic and protonated forms of the chromophore (Figure 3B). As expected, the fluorescence lifetime increased with increasing pH. Thus, in acidic conditions, due to the presence of a mainly protonated chromophore, the lifetime was approximately 1.2 ns, while, in an alkaline environment, where the anionic form of the chromophore dominates, the fluorescence lifetime increased to 2.4 ns. So, it is fundamentally possible to estimate pH by measuring the fluorescence lifetime of the SypHer3s sensor using the 445 nm or a similar laser as a source of excitation light. 

**Figure 4:**
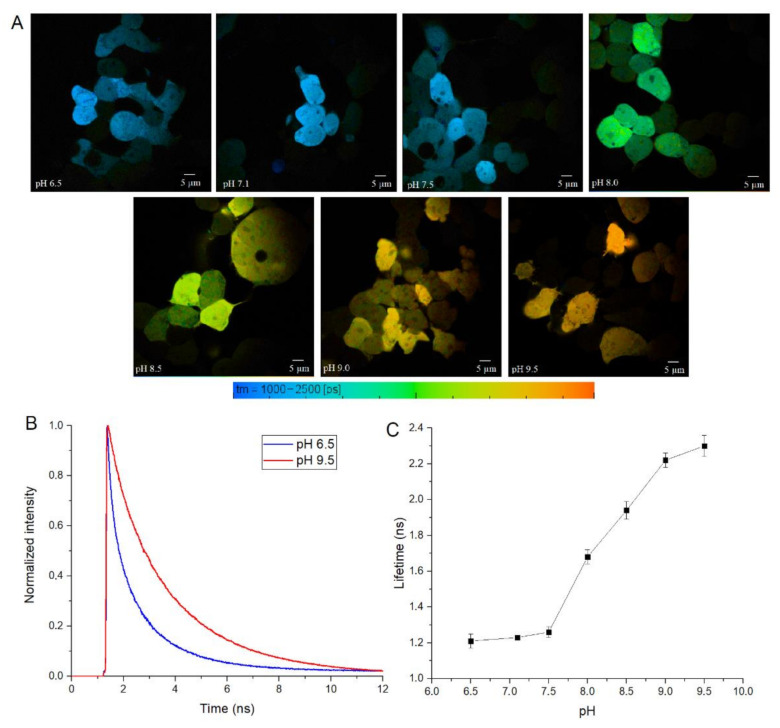
(**A**) FLIM images of the nigericin-treated HEK293 cells expressing cytoplasmic SypHer3s sensor at different pH values varying from 6.5 (upper-left image) to 9.5 (lower-right image); (**B**) fluorescence decay kinetics of the cytoplasmic SypHer3s in HEK293 cells treated with nigericin at pH 6.5 (blue curve) and pH 9.5 (red curve); (**C**) the pH dependence of the cytoplasmic SypHer3s fluorescence lifetime in HEK293 cells treated with nigericin. Values are the average of ≥4 different cells ± S.D.

To test SypHer3s in cellulo, we performed calibration experiments in HEK 293 cells, by treating them with nigericin ionophore and using a set of Tris-HCl buffers with a pH from 6.5 to 9.5. For these experiments, we used two-photon excitation at 890 nm, which should excite both the neutral and anionic forms of the fluorophore. We transfected HEK 293 cells with a construct that encodes cytoplasmic SypHer3s protein with eukaryotic expression, and treated these cells with antibiotic nigericin, which acts as a H^+^ and K^+^ ionophore, and therefore equalizes the pH value inside and outside the cell [23,24]. Then, we measured the fluorescence lifetime under different pHs (Figure 4A and Figure S3). The exposure of cytoplasmic SypHer3s to different pHs also shows the correlation between fluorescence lifetime and pH (Figure 4).

**Figure 5:**
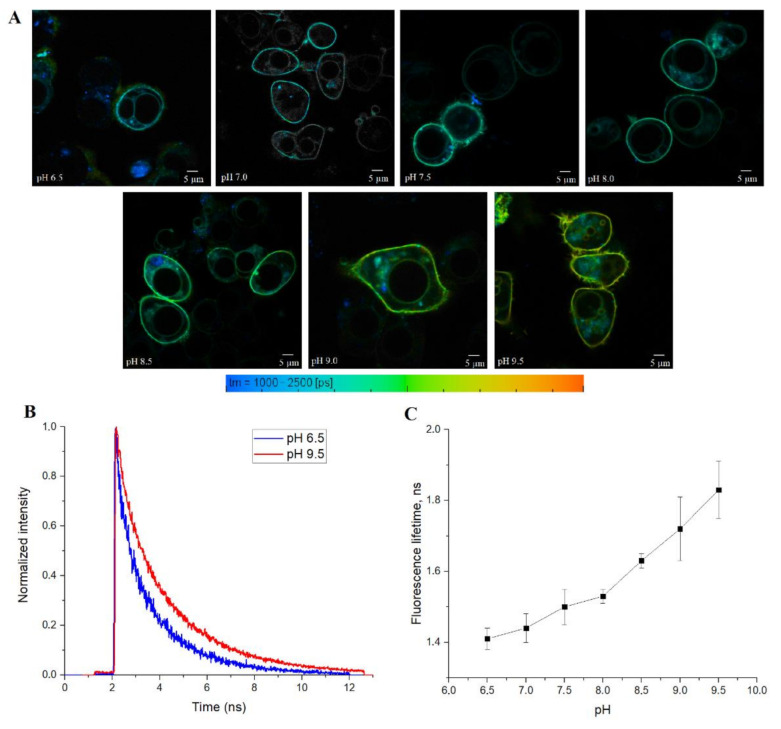
(**A**) FLIM images of the HEK293 cells expressing SypHerExtra sensor at different pH values from 6.5 (upper-left image) to 9.5 (lower-right image); (**B**) fluorescence decay kinetics of SypHerExtra in HEK293 cells at pH 6.5 (blue curve) and pH 9.5 (red curve); (**C**) the pH dependence of SypHerExtra fluorescence lifetime in HEK293 cells. Values are the average of ≥6 different cells ± S.D, except for pH 9.0, where only three cells were detected.

Finally, we tested SypHerExtra for measuring extracellular pH bound to the membrane in live cells. HEK 293 cells were transfected with this construct, and treated with Tris-HCl buffer with pH ranging from 6.5 to 9.5 (Figure 5A and Figure S4). The detection of the fluorescence lifetime of SypHerExtra by FLIM technique shows changes in the fluorescence lifetime, as in previous experiments (Figure 5B,C).

## 4. Discussion

Measuring pH in complex biological systems in vivo represents a significant biological challenge, especially when it comes to small animals, such as mice. pH-sensitive probes must not interfere with the natural environment and should be small enough to allow an accurate analysis of local pH variations. Genetically encoded fluorescent pH sensors are one of the best choices to achieve this goal [11].

Although the general paradigm is that the pH of blood and other extracorporeal liquids is close to neutral, there are examples of significant pH shifts in visceral organs, especially those that are involved in food digestion [25]. Also, a lot of experimental data have been accumulated that suggest the existence of pH local shifts in the nervous system [26].

Recently, we demonstrated that at least three receptor tyrosine kinases—insulin receptor-related receptor (IRR), ErbB2, and c-Met—can be activated by incubation with mildly alkaline extracellular medium with pH > 8.0 [27,28,29,30]. IRR is a receptor tyrosine kinase of the insulin receptor family, which can be activated merely by an increase in the extracellular pH (pH > 7.9), and in vivo experiments in knockout mice revealed the role of IRR as a regulator of bicarbonate excretion in the kidneys.

Since the known genetically encoded pH sensors are designed to measure intracellular pH, and most of them react to a mildly acidic environment, we aimed to generate a genetically encoded ratiometric pH sensor that is suitable for extracellular pH measurements in the pH range from 7.4 to 9.0, which could be used in poorly transparent organs, such as the kidneys or stomach.

To achieve this goal, we created the SypHerExtra sensor, on the basis of the recently described fluorescent pH sensor Sypher3s [15], fused with a signal peptide and the transmembrane region of neurexin I, which is a cell adhesion receptor of the brain [31]. We showed that SypHerExtra is transported to the cytoplasmic membrane, where it can sense mildly alkaline media. It should be noted that we used transient cell transfection; therefore, the expression of both SypHer3s and SypHerExtra proteins varies from cell to cell. According to our data, all the cells that express SypHerExtra responded to the change in pH of the media, but the intensity of the fluorescence signal that we measured depends on the transfection efficiency.

Using FLIM, instead of intensity-based measurements, allowed us to minimize the spectral bleed-through of two fluorescent forms of SypHer3s and so-called inner filter effects, which are absorption and scattering events that affect the fluorescence intensity, especially in non-transparent tissues and organs [17].

In addition, we have successfully used the two-photon excitation method, using a pulsed laser, with a wavelength of 890 nm, as the source of excitation light. Compared with single-photon excitation, this method has a greater signal penetration depth; according to various estimates, it ranges from 0.5 to 1.6 mm [32,33,34]. In addition, long-wave excitation light has less scattering, lower phototoxicity, and reduced photobleaching of fluorophores due to its lower photon energy. Finally, since the required energy of excitation light is achieved in a very small focal volume, there is no fluorescent signal in the tissues located above and below the focal plane, which allows increased resolution compared to single-photon confocal microscopy [35]. 

It is interesting to note that SypHerExtra has a shorter fluorescence lifetime at pH 9.5 than recombinant or cytoplasmic SypHer3s. To be precise, the lifetime of cytoplasmic SypHer3s changes from 1.2 ns at pH 6.5 to 2.4 ns at pH 9.5, while SypHerExtra lifetimes are about 1.4 ns at pH 6.5 and 1.8 ns at pH 9.5. In comparison with the cytoplasmic SypHer3s, the dynamic range of SypHerExtra is lower. Such a difference could be explained by the limitations in measurements and data processing, as the resolution of the FLIM images is only 512 x 512 pixels; therefore, only the outer part of the cell membrane cannot be chosen as the region of interest (ROI)—there is also the SypHerExtra population located at the intracellular part of the membrane, and even some parts of cytoplasmic sensor that are taken into account during the fluorescence lifetime calculation. It is known that only extreme alkalinization (pH > 8.5) could affect the intracellular pH [36]. This phenomenon was also observed in our data. For example, the treatment of cells with a buffer of pH 9.5 resulted in an increase in the intracellular pH value to about pH 8.0 (Figure S3G). Also, the differences in lifetimes can be explained by the existence of two populations of SypHerExtra—the “truly” extracellular one, which has a fluorescence lifetime corresponding to the buffer pH, and another one that seems intracellular, which is located inside the cell endosomes and has a lower fluorescence lifetime related to the intracellular endosomal pH. The resulting fluorescence lifetime for these two populations is lower than the SypHer3s fluorescence lifetime at the same pH. Nevertheless, there is a strong pH dependence of fluorescence lifetime for both the cytoplasmic SypHer3s protein and the extracellular membrane-bound SypHerExtra sensor that allows intracellular and extracellular pH values to be measured in living cells by the FLIM method.

## Supplementary Materials

The following are available online at https://www.mdpi.com/article/10.3390/bios11090340/s1, Figure S1: Fluorescent images of the fixed HeLa cells expressing SypHerExtra sensor, Figure S2. FLIM images of the nigericin-treated HEK293 cells expressing cytoplasmic SypHer3s sensor at different pH values: (A) pH 6.5; (B) pH 7.1; (C) pH 7.5; (D) pH 8.0; (E) pH 8.5; (F) pH 9.0; (G) pH 9.5, Figure S3. FLIM images of the HEK293 cells expressing SypHerExtra sensor at different pH values: (A) pH 6.5; (B) pH 7.0; (C) pH 7.5; (D) pH 8.0; (E) pH 8.5; (F) pH 9.0; (G) pH 9.5, Figure S4. Fluorescent images of the HEK293 cells expressing SypHerExtra sensor at pH 8.5 (A) without nigericin and (B) after incubation with nigericin.
