# Supplementary material for: FLIM-Based Intracellular and Extracellular pH Measurements Using Genetically Encoded pH Sensor
Source: Biosensors (Basel). 2021 Sep 15;11(9):340. doi: 10.3390/bios11090340 (PMC8468847; doi:10.3390/bios11090340)
Supplement: Supplementary file 1 [file biosensors-11-00340-s001.zip › biosensors-1355084-supplementary.pdf]

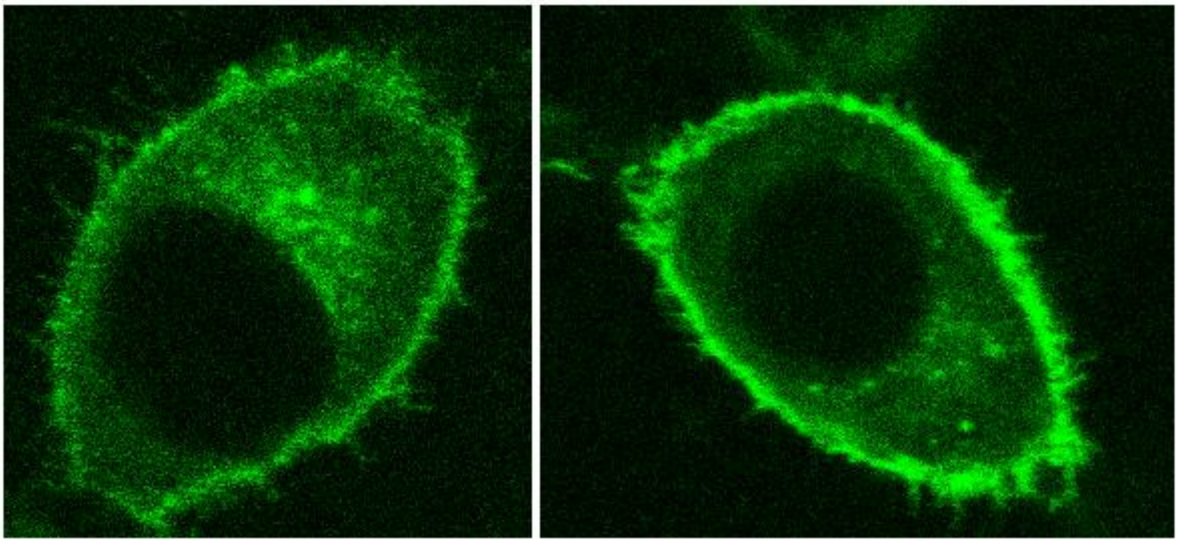

Figure S1. Fluorescent images of the fixed HeLa cells expressing SypHerExtra sensor.

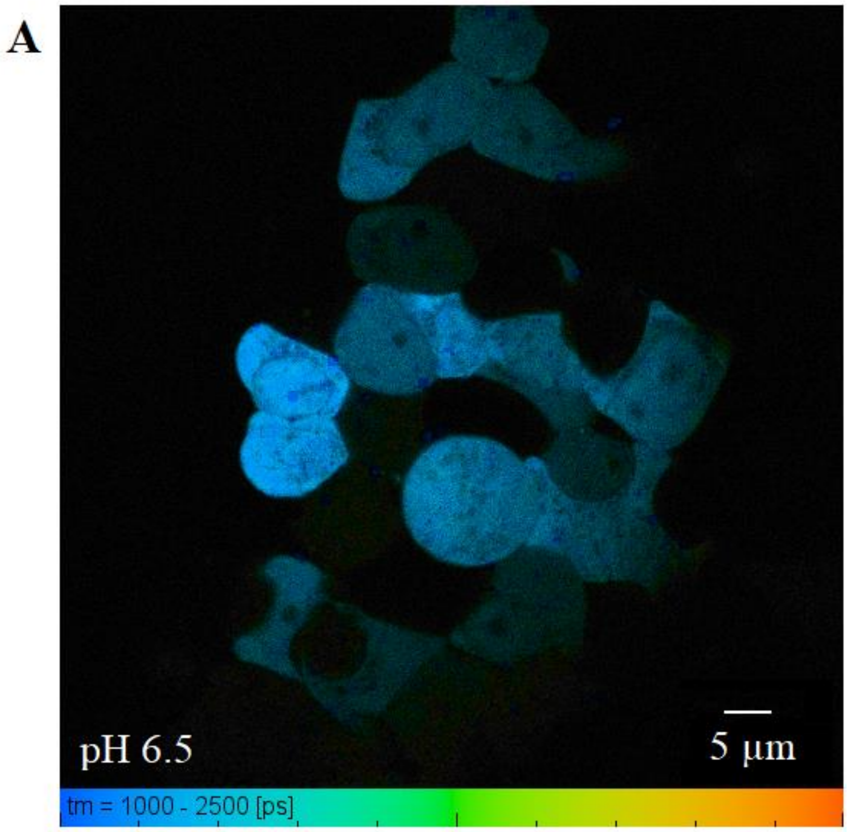

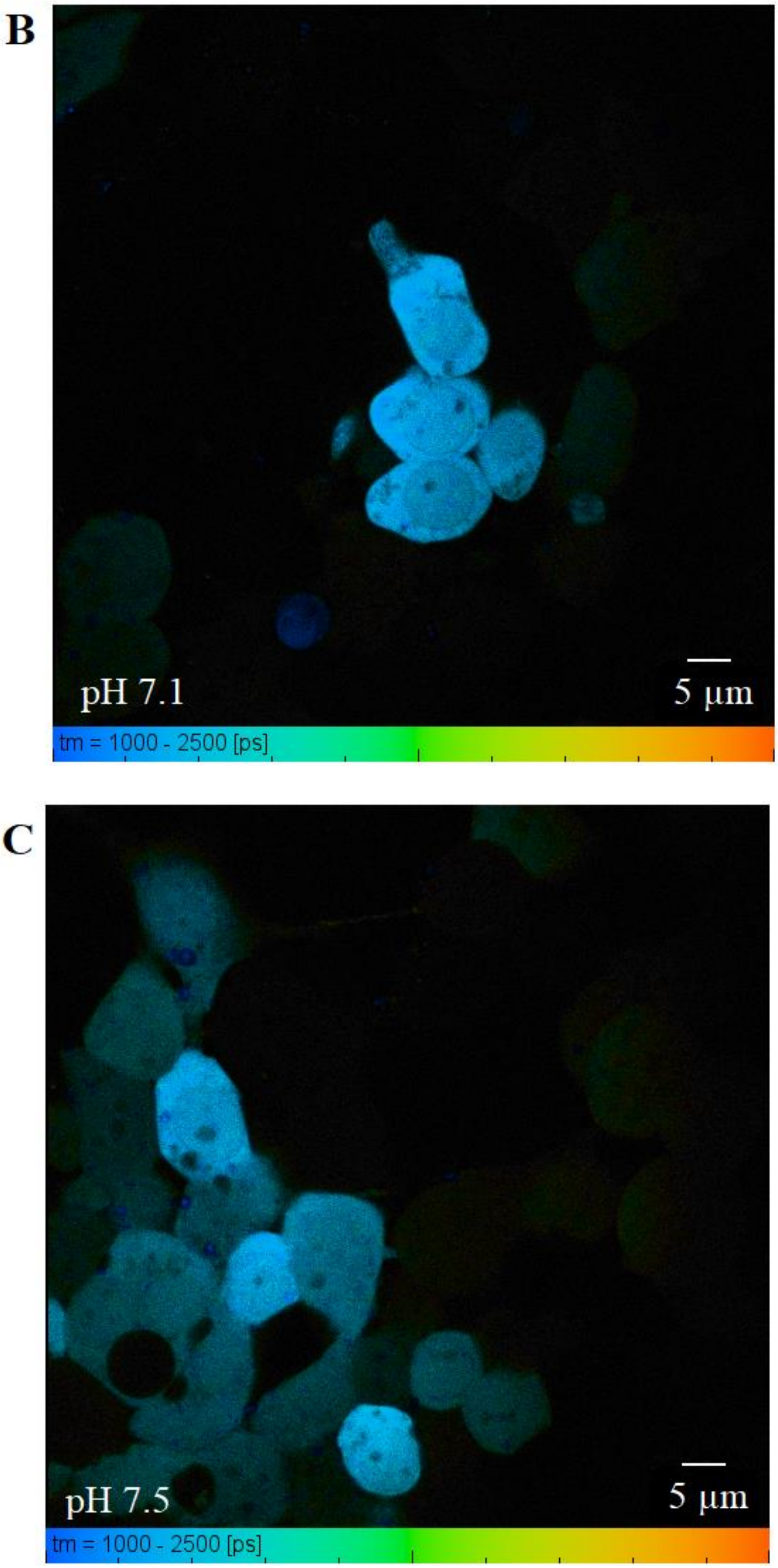
B
pH 7.1
5 µm
tm = 1000 - 2500 [ps]
C
pH 7.5
5 µm
tm = 1000 - 2500 [ps]

D

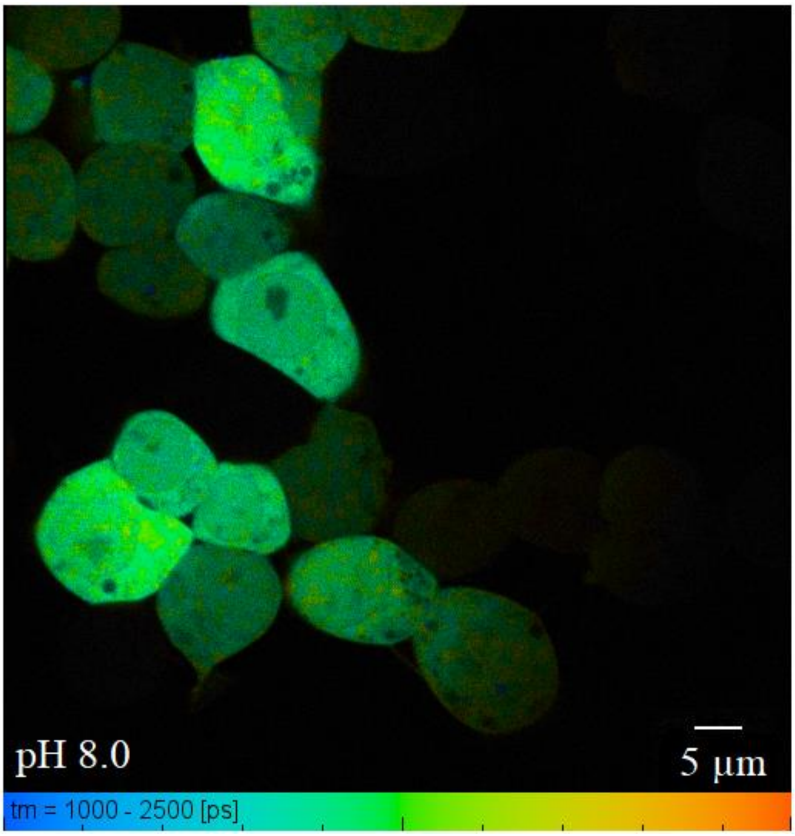

E

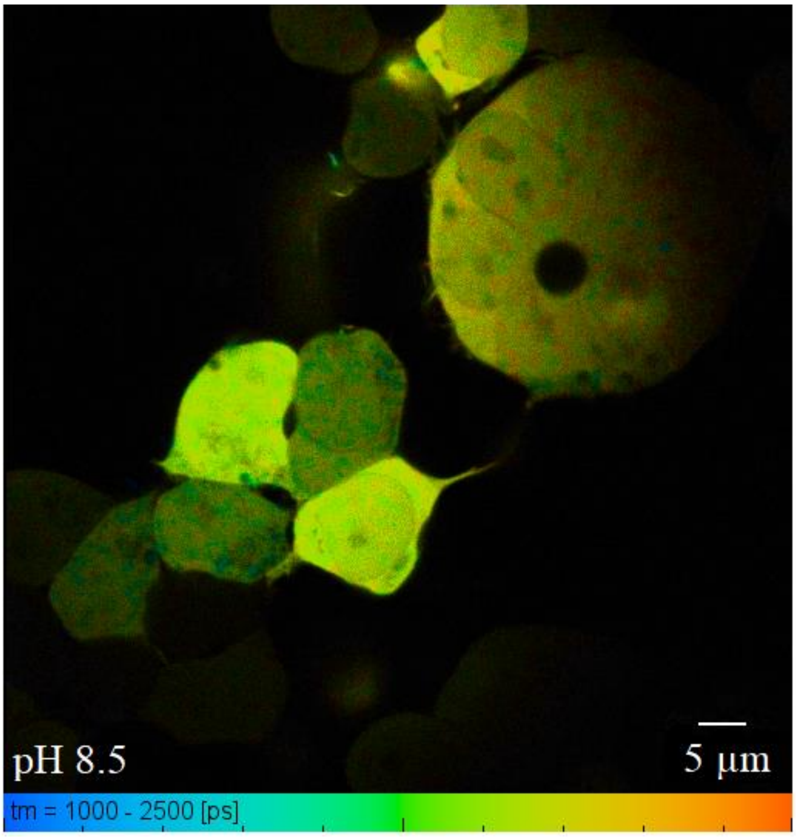

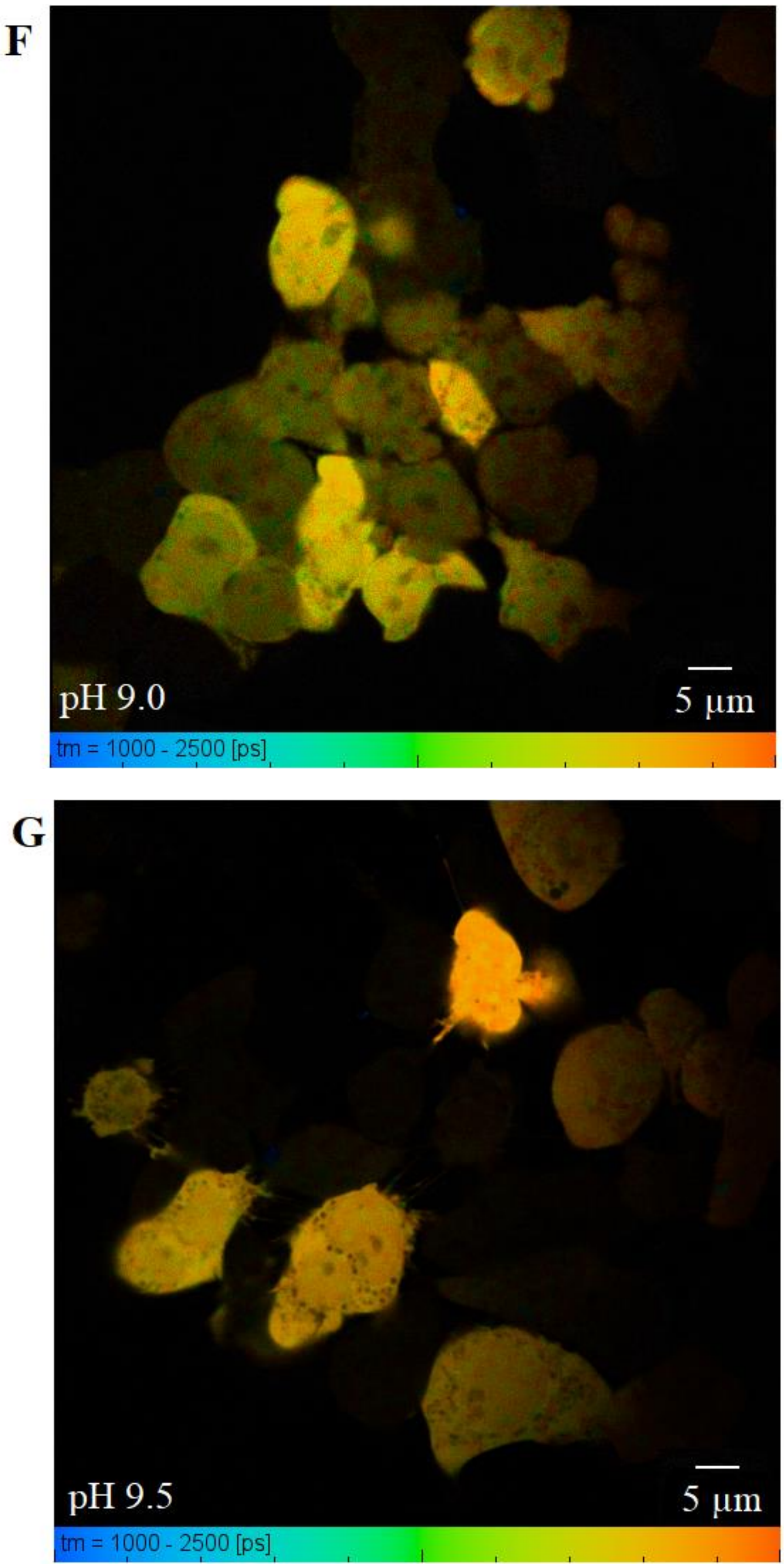

Figure S2. FLIM images of the nigericin-treated HEK293 cells expressing cytoplasmic SypHer3s sensor at different pH values: **(A)** pH 6.5; **(B)** pH 7.1; **(C)** pH 7.5; **(D)** pH 8.0; **(E)** pH 8.5; **(F)** pH 9.0; **(G)** pH 9.5;

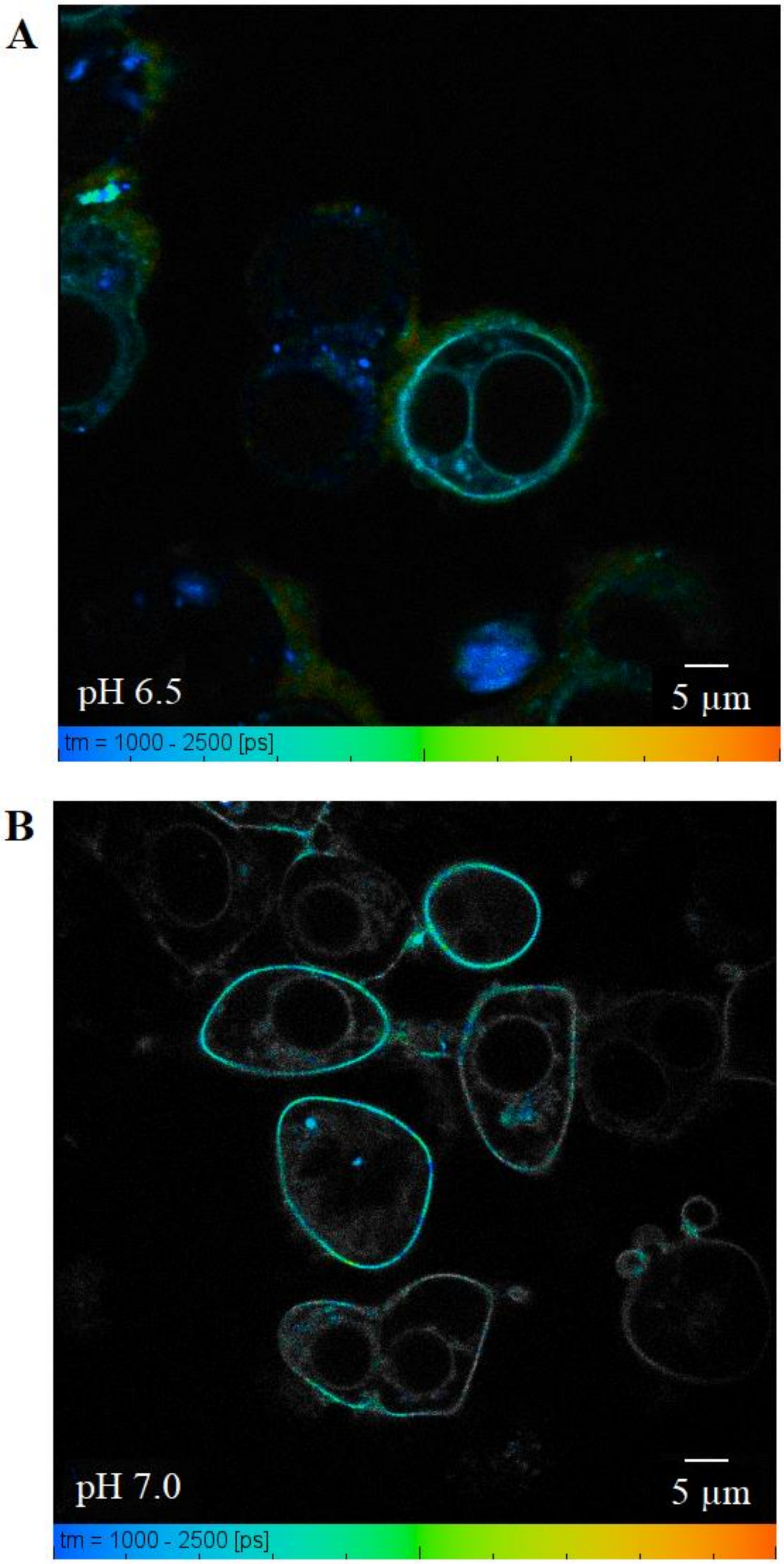
A
pH 6.5
5 µm
tm = 1000 - 2500 [ps]
B
pH 7.0
5 µm
tm = 1000 - 2500 [ps]

C

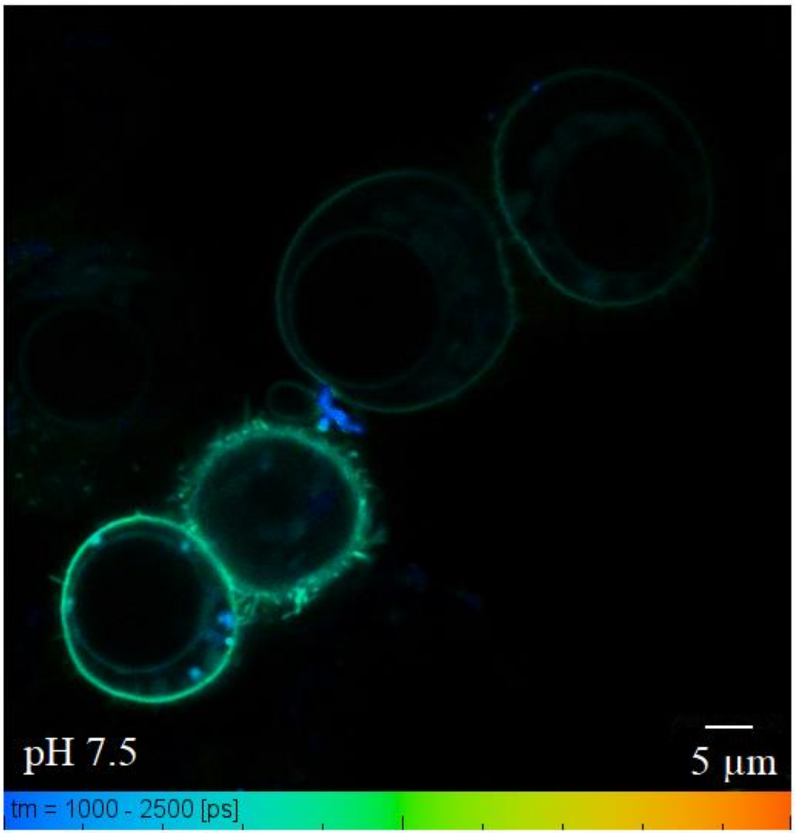

D

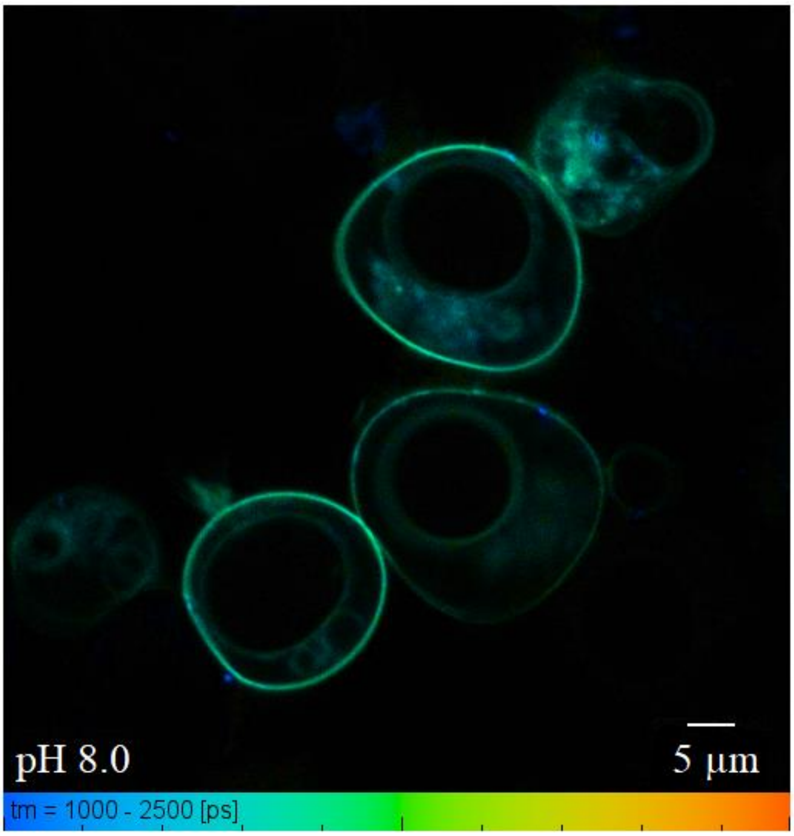

E

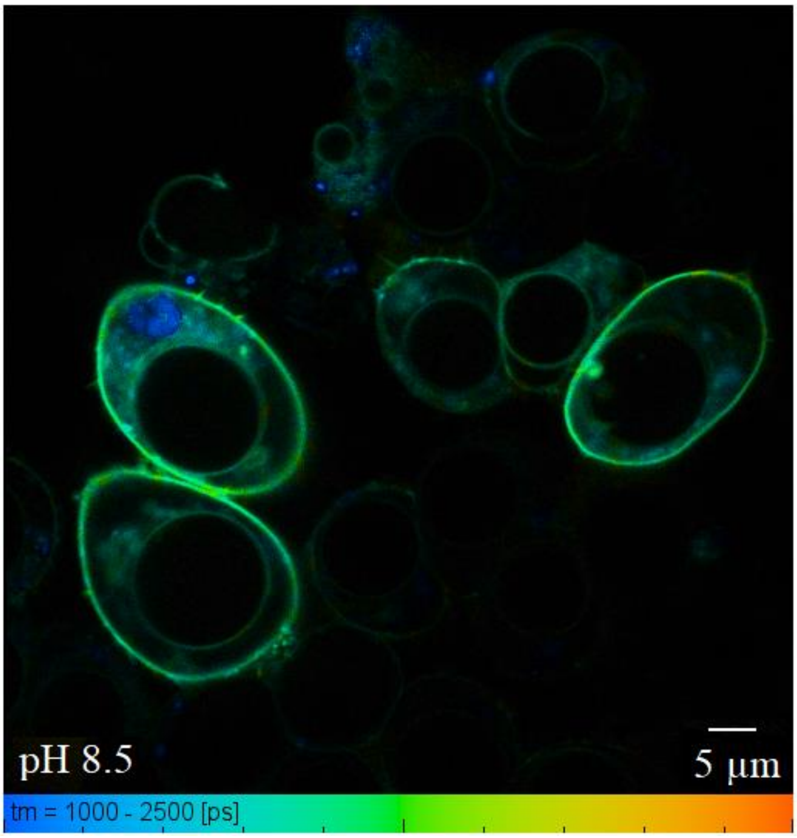

F

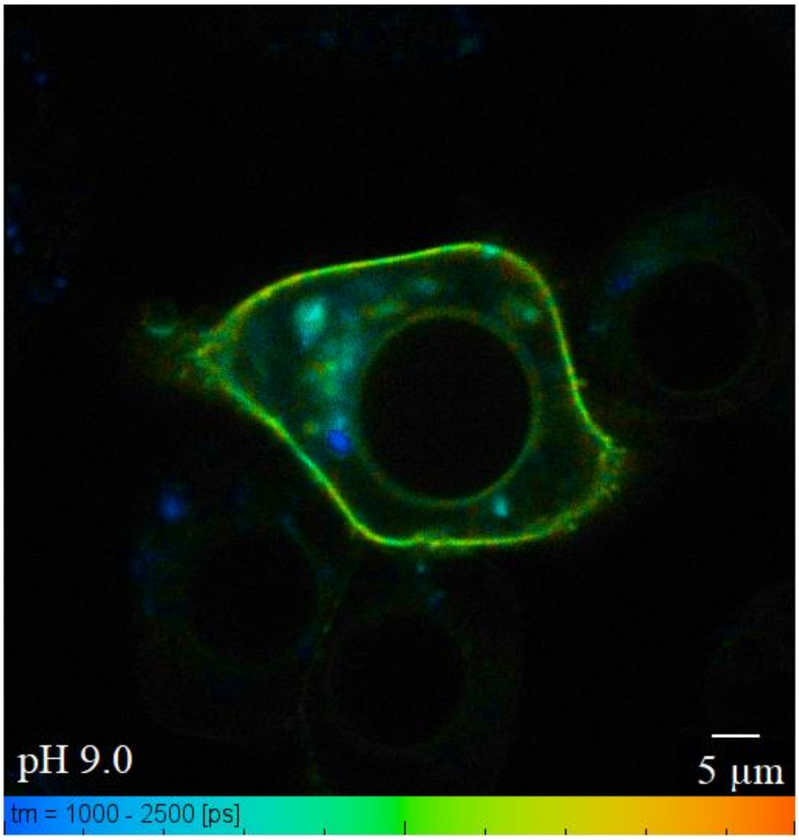

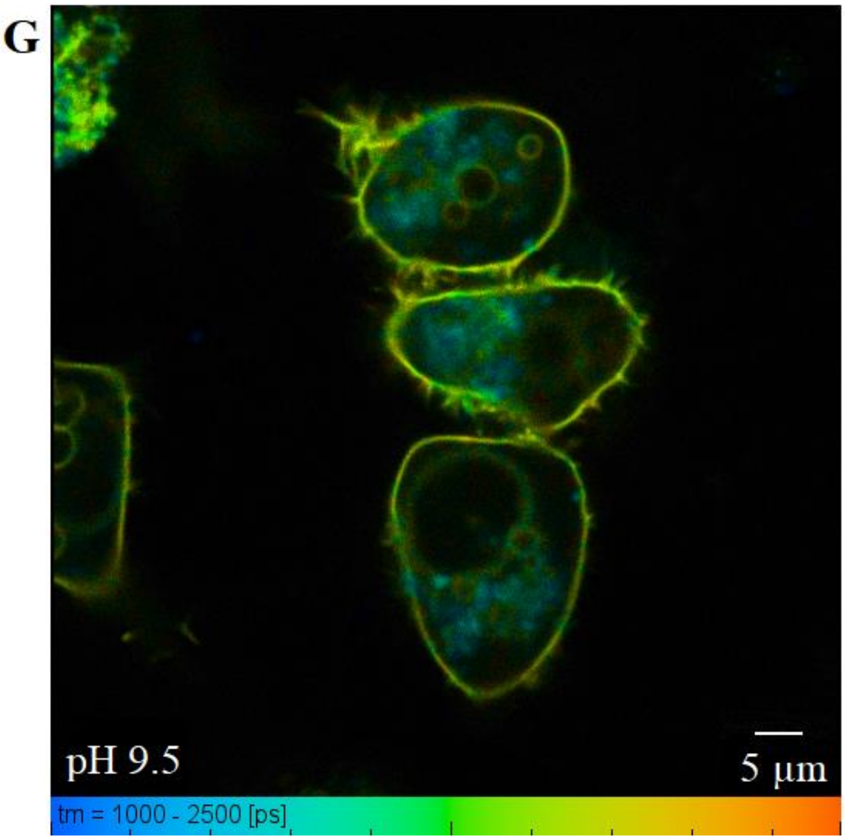

Figure S3. FLIM images of the HEK293 cells expressing SypHerExtra sensor at different pH values: **(A)** pH 6.5; **(B)** pH 7.0; **(C)** pH 7.5; **(D)** pH 8.0; **(E)** pH 8.5; **(F)** pH 9.0; **(G)** pH 9.5;

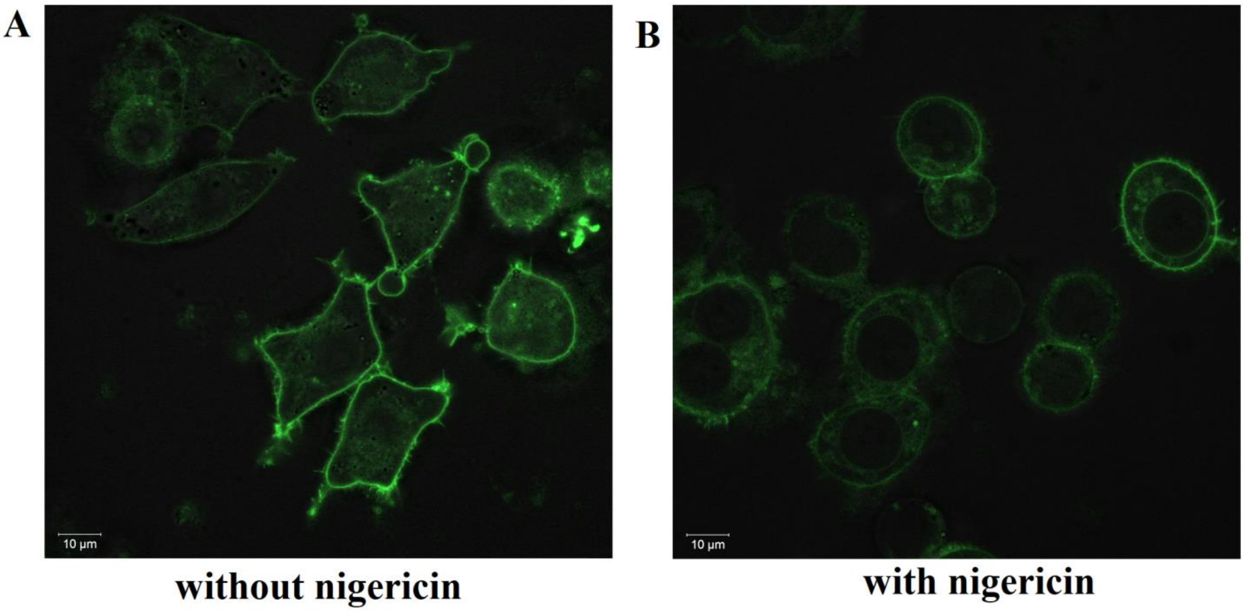

Figure S4. Fluorescent images of the HEK293 cells expressing SypHerExtra sensor at pH 8.5 **(A)** without nigericin and **(B)** after incubation with nigericin.
